# Enlarged Virchow Robin spaces associate with cognitive decline in multiple sclerosis

**DOI:** 10.1371/journal.pone.0185626

**Published:** 2017-10-18

**Authors:** Alice Favaretto, Andrea Lazzarotto, Alice Riccardi, Stefano Pravato, Monica Margoni, Francesco Causin, Maria Giulia Anglani, Dario Seppi, Davide Poggiali, Paolo Gallo

**Affiliations:** 1 Multiple Sclerosis Centre of the Veneto Region, Department of Neurosciences, University Hospital of Padova–Medical School, Padova, Italy; 2 Neuroradiology Unit, Azienda Ospedaliera di Padova, Padova, Italy; NIH Clinical Center, UNITED STATES

## Abstract

The clinical significance of Virchow Robin spaces (VRS) in inflammatory brain disorders, especially in multiple sclerosis (MS), is still undefined. We analysed enlarged VRS (eVRS) by means of phase sensitive inversion recovery (PSIR) MRI sequence and investigated their association with inflammation or brain atrophy, and to clinical or physical disability. Forty-three MS patients (21 clinically isolated syndrome suggestive of MS [CIS], 15 RRMS, 7 progressive [PMS]) and 10 healthy controls (HC) were studied. 3DT1, 3DFLAIR and 2DPSIR images were obtained with a 3T MRI scanner. eVRS number and volume were calculated by manual segmentation (ITK-SNAP). Freesurfer was used to assess brain parenchymal fraction (BPF). All patients underwent clinical (EDSS) and cognitive (Rao’s BRB and DKEFS) evaluation.

eVRS number and volume resulted significantly higher on 2D-PSIR compared to both 3D-T1 (p<0.001) and 3D-FLAIR (p<0.001) and were significantly increased in CIS compared to HC (p<0.05), in PMS and RRMS compared to CIS (p<0.001) and in male versus female patients (p<0.05). eVRS volume increased significantly with disease duration (r = 0.6) but did not correlate with EDSS. eVRS significantly correlated with SPART*d* (r = -0.47) and DKEFS*fs* (r = -0.46), especially when RRMS and PMS were merged in a single group (r = 0.89, p = 0.002 and r = 0.66, p = 0.009 respectively), while no correlation was found with BPF (r = 0.3), gadolinium-enhancing lesions (r = 0.2) and WMT2 lesion volume (r = 0.2). 2DPSIR allowed the detection of an impressive higher number of eVRS compared to 3DT1 and 3DFLAIR. eVRS associate with SPART*d* and DKEFS*fs* failure in relapse-onset MS, suggesting they may contribute to cognitive decline in MS.

## Introduction

The perivascular compartments surrounding small cerebral blood vessels, called Virchow–Robin spaces (VRS), are fluid-filled spaces that become visible on MRI after a substantial increase in volume [1]. Enlarged VRS (eVRS) can especially be observed in three characteristic locations: along the lenticulostriate arteries entering the basal ganglia (BG), along the arteries entering the cortical grey matter over the high convexities and extending into the white matter, and in the midbrain [1]. Traditionally, eVRS have been thought to be a casual MRI finding lacking pathological significance or related to brain senescence. However, several observations have questioned this view and suggested their possible role as magnetic resonance imaging (MRI) marker for neurodegenerative brain pathologies [2–6]. Indeed, although eVRS are more frequently detected with increasing age [1,7], a significant increase in their number and volume was reported in vascular or neurodegenerative diseases [8–13]. Moreover, since one of the most common locations for eVRS are the basal ganglia and the ponto-mesencephalic junction in the midbrain, an association with extrapyramidal disorders has also been suggested [10, 14].

In multiple sclerosis (MS), eVRS have been associated with either white matter (WM) [6,15] or grey matter (GM) pathology [16] as disclosed by MRI. However, the few studies available in the literature showed conflicting results. Discrepancies are probably due to the low number of patients enrolled in the studies, patients age, substantial differences in both the methodologies and the strength of the MRI field applied (i.e., 1.5 vs 3.0 vs 7.0 tesla).

In previous studies on MS cortical lesions [17,18] we incidentally observed higher numbers of eVRS on phase sensitive inversion recovery (PSIR) images compared to T1 and FLAIR images. Thus, we designed a study aimed at analysing, by means of PSIR, the number and volume of eVRS in MS patients, as well as their association with MRI parameters of inflammation (gadolinium-enhanced lesions and T2 white matter lesion volume) or neurodegeneration (brain atrophy) and with physical or cognitive disability.

## Materials and methods

### Patients

Forty-three patients, whose diagnosis was achieved according to the McDonald/Polman’s diagnostic criteria [19], and ten gender and age matched healthy controls (HC) were enrolled in the study. Neurological examination was performed and the Expanded Disability Status Scale (EDSS) was calculated. In order to obtain a comprehensive view of eVRS number and volume across different disease stages, the patients were selected to comprise a wide range of disease duration (0.33–41.5 years) and disability (EDSS range: 1.0–7.0). Twenty-one patients had the diagnosis of clinically isolated syndrome (CIS) suggestive of MS (i.e., all patients had dissemination in space of lesions and the presence of IgG oligoclonal bands in the cerebrospinal fluid), 15 had relapsing remitting MS (RRMS) and 7 had progressive MS (PMS). No patient had cardiovascular diseases or risk factors for ischemic brain disorders, nor the evidence of other neurodegenerative or inflammatory diseases. No patient had been treated with high dose steroids in the month prior to MRI examination. The study was approved by the local Ethics Committee (Comitato Etico per la Sperimentazione, Azienda Ospedaliera–Università degli Studi di Padova). All patients gave written informed consent.

### Neuropsychological and neurological evaluations

The Rao’s Brief Repeatable Battery of Neuropsychological Tests (BRB-NT) and the Delis-Kaplan Executive Function System Sorting test (D-KEFS ST) were administrated by an expert neuropsychologist (AR) in agreement with the procedure referred to the original manuals and the normative data for Italian population [20–21]. The neurological examination was performed by means of the Expanded Disability Status Scale (EDSS).

### MRI examination

Images were acquired using a 3T scanner (Ingenia, Philips Medical Systems, Best, The Netherlands) with 33 mT/m power gradient and a 64-channel head coil. No major hardware upgrades occurred during the study, and bimonthly quality-assurance sessions assured measurement stability. The following images were acquired for each subject: (a) 3DT1: repetition time (RT) 7.8 ms; echo time (ET) 3.6 ms; 180 contiguous axial slices with the off-center positioned on zero with thickness of 1.0 mm; flip angle = 8°; matrix size = 220×220; FOV = 220×220×180 mm3. (b) 3D-FLAIR: RT 4800 ms; ET 310 ms; inversion time (IT) 1650 ms; 365 contiguous axial slices with thickness of 1.0 mm; matrix size 256×256; and FOV = 256×256×182 mm3; c) 2D-PSIR: resolution 1×1×3 mm, FOV 230×200 mm, RT 7000 ms, ET 13 ms, IT 400 ms, 40 slices, time 7 mins. eVRS were identified separately on 3D-T1, 3D-FLAIR and 2D-PSIR by three independent examiners (AF, DP, PG). The intraobserver agreement (i.e., the same MRI scans were analysed three times by the same examiner) and inter-observer agreement (i.e., three independent observers examined the images of five patients) in computing eVRS number and volume were calculated and were settled at >95% and >90% (i.e., almost perfect agreement), respectively. eVRS number and volume were calculated by manual segmentation (ITKSNAP). Brain parenchymal fraction (BPF) was assessed by means of Freesurfer. On the base of size and volume of the rare VRS detectable in the brain of HC, VRS were define ‘enlarged’ when their diameter was >2mm and their volume >2 mm^3^. Volume of eVRS was computed as the number of voxels in VRS VOI multiplied by the volume of a voxel, as indicated in the image metadata.

**Fig 1:**
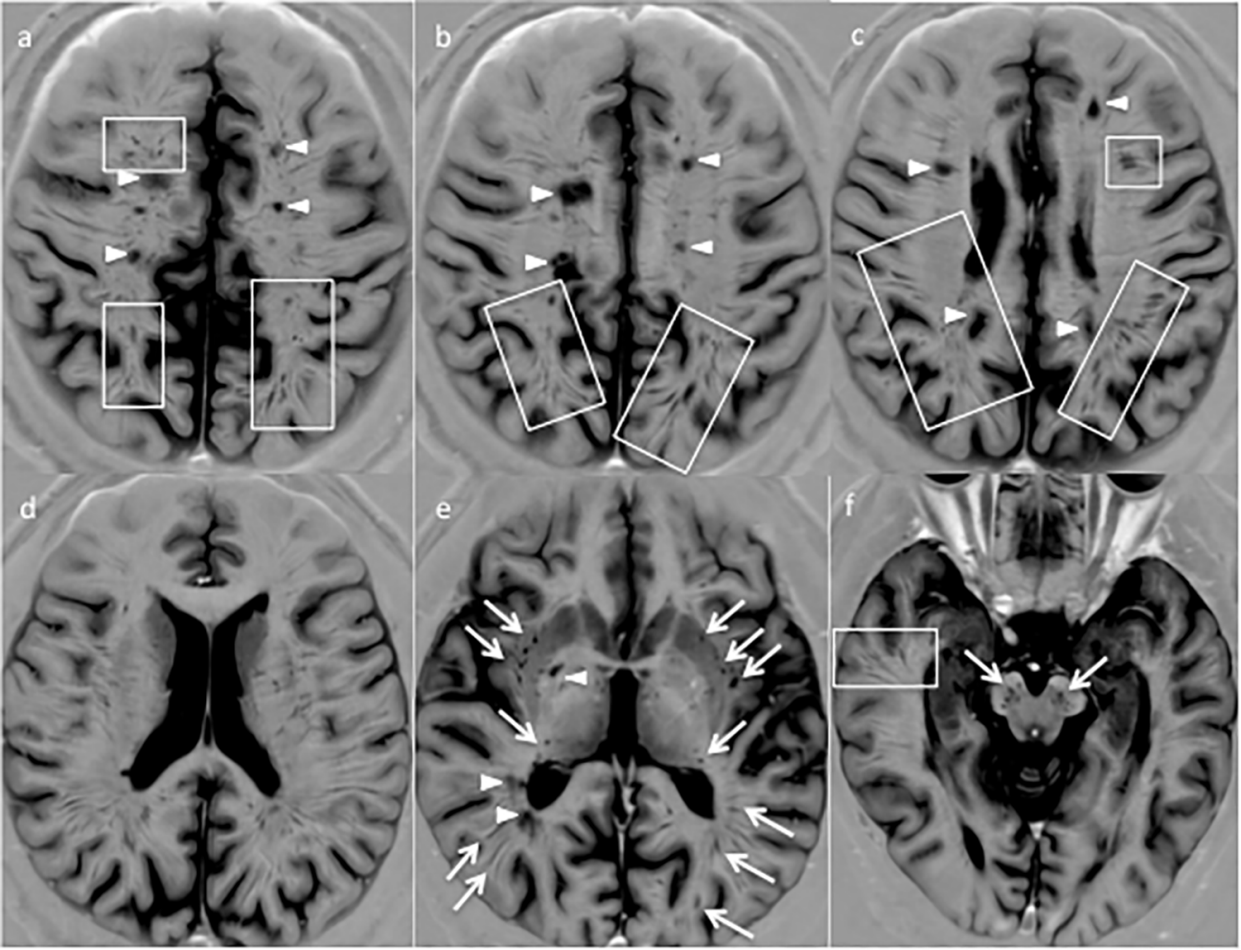
Phase sensitive inversion recovery (PSIR) images of a brain of a relapsing-remitting MS patient disclose an unexpected high number of eVRS. An impressive high number of eVRS can be observed throughout the white matter of both hemispheres, in the basal ganglia (e) and in the brainstem (f) (arrows). eVRB usually have a round-shape in the basal ganglia and in the brainstem, while they appeared ovoid-shaped or stripe-like in the hemisphere’s white matter.

**Fig 2:**
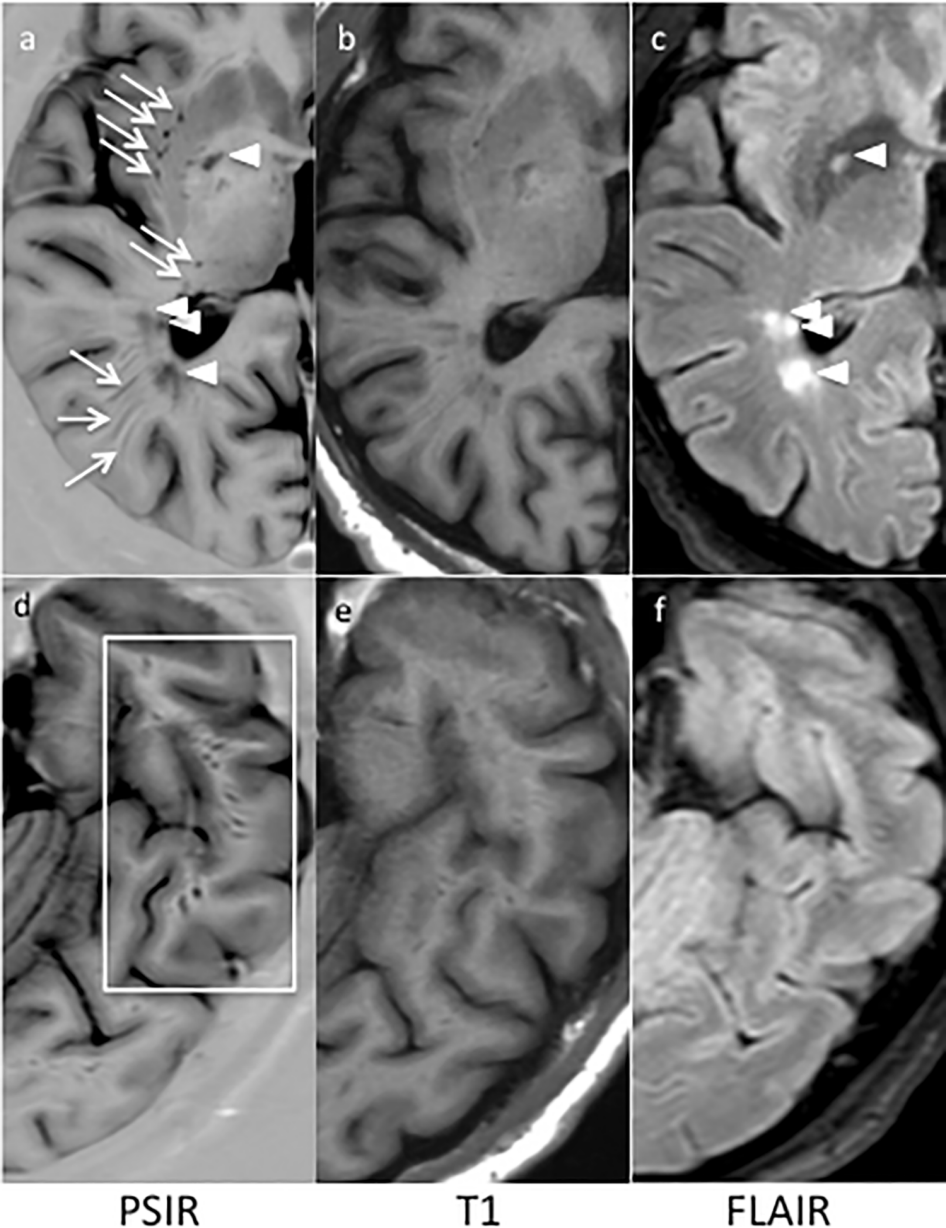
**The comparison of PSIR (a,d), T1 (b,e) and FLAIR (c,f) images from a brain of a RRMS patient clearly discloses the superiority of PSIR in detecting eVRS.** In (a) eVRS are indicated by arrows while WM lesions are indicated by arrow heads (see the corresponding hyperintense lesions on FLAIR image (c)). In (d), at least 10 eVRS can be counted inside the white rectangular frame, but are hardly identified on the corresponding T1 (e) or FLAIR (f) images. The discrimination between eVRS and MS lesions is easily done by comparison of PSIR and FLAIR sequences.

**Fig 3:**
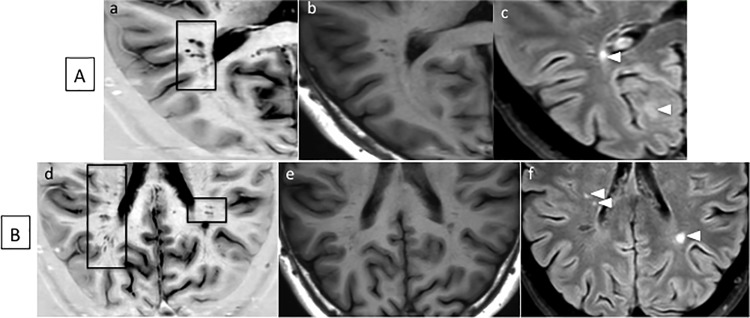
**PSIR (a,d), T1 (b,e) and FLAIR (c, f) images of eVRS of two CIS patients (A, B). A high number of eVRS can be detected by PSIR even in patient at clinical onset and having low WM lesion load.** Arrow heads indicate WM lesions. On FLAIR images (c,f) eVRS are easily distinguished from inflammatory lesions (hyperintense) for their hypointense signal. In patient (A), a cluster of at least five round-shaped eVRS is clearly visible on PSIR image (a, black rectangular frame) and is, although less clearly, visible on T1 and FLAIR images. In patient (B), many eVRS of various shapes can be observed on PSIR (d) while only a few of them are visible on T1 (e) or FLAIR (f) images, thus further confirming the superiority of PSIR in detecting eVRS.

**Fig 4:**
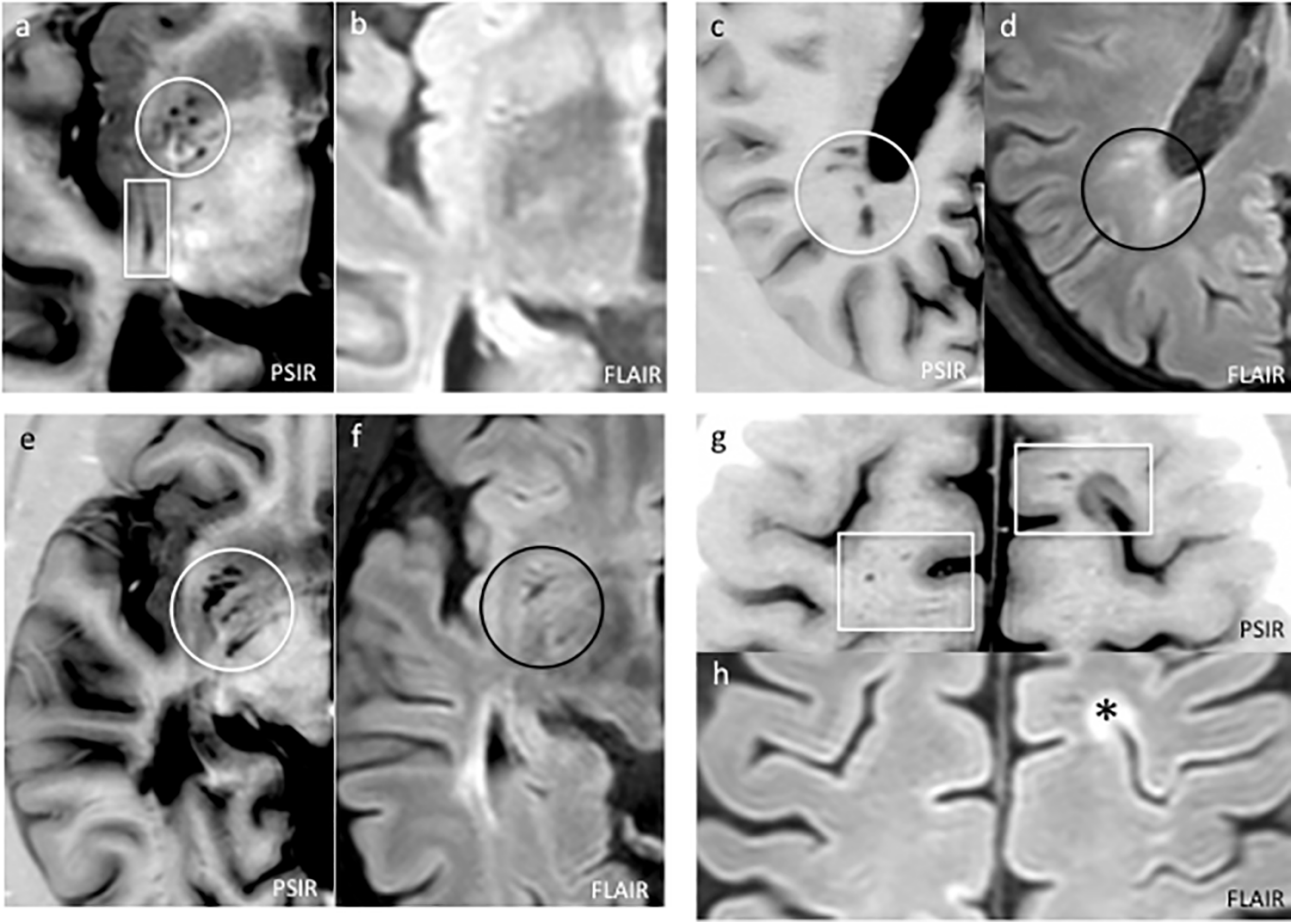
**The comparison of PSIR (a,c,e,g) and FLAIR (b,d,f,h) images allows a clear discrimination of eVRS from white matter and grey matter lesions.** In (a) and (b) several round-shaped (circle) and one stripe-like (rectangle) eVSR are clearly identified on PSIR, while on the FLAIR image they can hardly be recognised. A cluster of small inflammatory lesions that appear hypointense in PSIR and hyperintense in FLAIR are visible in (c) and (d). The comparison of PSIR and FLAIR images is necessary to discriminate small inflammatory lesions from eVRS. Several eVRS can be observed in the basal ganglia in (e) and (f): also in this case PSIR significantly improves the evaluation of their number and volume. In (g) and (f) some eVRS (rectangles) are observed in the convexity of a CIS patient (* indicates a mixed white/grey matter lesion).

**Fig 5:**
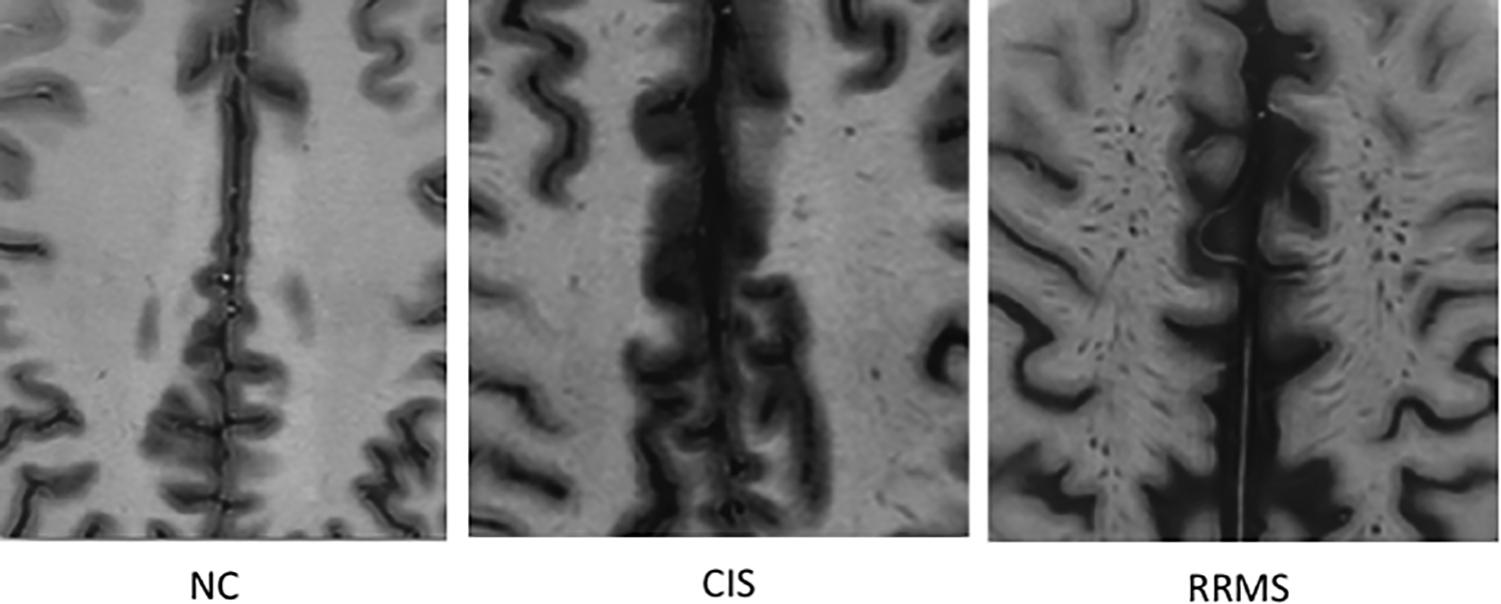
The number and volume of eVRS are higher in RRMS patients compared to age-matched CIS patients. PSIR images showing the WM of the convexities of a normal control (NC) subject, a patients with a clinically isolated syndrome (CIS) suggestive of MS (the patients had dissemination in space of T2 lesions and the presence of oligoclonal IgG in the CSF) and a relapsing-remitting MS patients. The three subjects were matched for age. While eVRS are not observed in HC, their number and volume were significantly higher in RRMS compared to CIS.

The MRI features of VRS have been described in detail by Kwee and Kwee (2007) and Etemadifar et al. (2011). Since VRS represent entrapments of interstitial fluid, they appear as hypo-intense lesions (spots/stripes) with all pulse sequences, thus they can easily be distinguished from MS WM lesions on FLAIR images. The brain parenchyma surrounding VRS generally has normal signal intensity in all the sequences used. Since eVRS develop around penetrating vessels, T1-weighted images with substantial flow sensitivity may show high signal intensity due to inflow effects, thereby helping to confirm that one is indeed dealing with VR spaces. On the base of their shape, location and signals on T1 and FLAIR images, eVRS can easily be differentiated from other types of lesion, such as lacunar infarctions, cystic periventricular leukomalacia, inflammatory lesions, cryptococcosis, mucopolysaccharidosis, arachnoid cysts and neuroepithelial cysts. Moreover, eVRS cannot be confused with normal vessels since these are not visible on T1, FLAIR and PSIR images of HC brain (see Figs 1–5). In other words, the enlargement of VRS implies the dilatation of the CSF-containing space around vessels.

### Statistics

Wilcoxon signed-rank test was used to compare 2DPSIR, 3DT1-weighted and 3DFLAIR eVRS counts. EDSS and age of each clinical subtypes was compared with Mann–Whitney U Test. Correlations between eVRS number or volume with EDSS, disease duration, and neuropsychological parameters were evaluated with Spearman’s rank correlation coefficient. SPSS Statistics 20 was used to perform the statistical analysis, and *p*<0.05 was considered significant.

## Results

### Patient characteristics

The 21 CIS patients had a mean disease duration <3 years (range: 0.1–3.0) and mean EDSS = 1.6±0.4, the 15 RRMS had a mean disease duration of 8.7±5.9 years (range 0.2–18) and mean EDSS = 2.2±0.6, and the 7 patients with PMS had a mean disease duration of 13.9±7.4 years (range 8–26) and mean EDSS = 6.0±1.5. The three groups of patients did not significantly differed in age (mean age: CIS = 36.3±9.3 years, range 17–52, RRMS = 35.3±9.1, range 17–55, and PMS = 41.3± 11.0, range 27–56; p>0.05 for all comparisons). The demographic and clinical features of the patients are summarized in Table 1.

**Table 1:** Demographic and clinical features of patients and healthy controls included in the study.

	F/M	Age	Disease Duration	EDSS
CIS	16/5	36.3±9.3 [17–52]	1.0±0.8 [0.1–3]	1.6±0.4 [1.0–2.5]
RRMS	7/8	35.3±9.1 [17–55]	8.7±5.9 [0.2–18]	2,2±0.6 [1.5–3.5]
PMS	4/3	41.3±11.0 [27–56]	13.9±7.4 [8–26]	6.0±1.5 [2.5–7.5]
Total patients	27/16	36.9±9.8 [17–56]	5.6±7.1 [0.1–26]	2.5±1.8 [1.0–7.5]
HC	6/4	33.0±8.6 [18–46]	-	-

Age and disease duration are expressed in years as mean±standard deviation (range into brackets). CIS = clinically isolated syndrome, RRMS = relapsing remitting multiple sclerosis, PMS = progressive MS. EDSS: Expanded Disability Status Scale. HC: Healthy Controls.

### VRS number and volume on PSIR, T1 and FLAIR

The number of VRS observed on 2DPSIR (as above mentioned, 3DPSIR was not available when this study was initiated) was significantly higher compared to that observed on both 3DT1 (p<0.001) and 3DFLAIR (p<0.001) images, being 3DT1 clearly superior to 3DFLAIR (p = 0.0002) (Table 2). The limited number of HC included in the study should not be considered a limitation since eVRS were only rarely observed in HC and only in the basal ganglia where they showed a round-shaped morphology. In some RRMS and PMS patients, the number of VRS observed on 2DPSIR images was unexpectedly and extraordinary higher compared to the number scored both on 3DT1 and 3DFLAIR (Fig 1). Indeed, many eVRS were observed on 2DPSIR in areas of apparently normal white matter on 3DT1 and 3DFLAIR images (Figs 2–4). The brain parenchyma surrounding VRS generally had normal signal intensity in all the sequences used.

**Table 2:** Volumes and numbers of eVRS obtained on PSIR, T1 and FLAIR images.

	eVRS volume (mm^3^)	eVRS number
**PSIR**	2783.96±2631.70 *	92.8±62.7 **
**T1**	490.56±621.38 **^**	16.7±20.7 **^^**
**FLAIR**	139.00±155.5	5.06±4.81

This is Table 2 legend. Volumes and numbers of eVRS are expressed as mean±standard deviation.

* = PSIR vs T1: p<0.00001, PSIR vs FLAIR: p<0.00001

** = PSIR vs T1: p<0.00001, PSIR vs FLAIR: p<0.00001

^ = T1 vs FLAIR: p = 0–0002

^^ T1 vs FLAIR: p = 0.0002

In order to compare our findings with the most relevant literature data, we focus our analysis only on eVRS, defined, by comparison with the VRS observed in HC subjects, as VRS having a diameter >2 mm and a volume >3 mm^3^ (Figs 2–4). The *number* of eVRS was still significantly higher on 2DPSIR images (mean number: 92.8±62.7) compare to that obtained with 3DT1 (mean number 16.7±20.7; p<0.00001) and 3DFLAIR (mean number 5.06±4.81; p<0.00001) images. Consequently, eVRS *volume* calculated on 2DPSIR images was also significantly higher (mean: 2783.96±2631.70 mm^3^) compared that calculated on 3DT1 (mean: 490.56±621.38 mm^3^, p<0.00001) or 3DFLAIR images (mean 139.00±155.5 mm^3^, p<0.00001).

From a morphological point of view, according to the direction of the vessels on the MRI section, we confirm that eVRS appear in three different shapes: 1) round-shaped, observed especially in the brainstem and in the midbrain, 2) ovoid-shaped, located primarily in periventricular regions and in basal ganglia, 3) stripe-like shaped, in the WM and the convexities of the hemispheres (Figs 2–4).

### eVRS across disease types

2DPSIR disclosed a significant increase in eVRS number and volume in CIS compared to HC (p<0.05) and in SPMS and RRMS compared to CIS (p<0.001)(Fig 5).

### Correlation analysis

The volume of eVRS obtained on 2DPSIR (but not on 3DT1 and 3DFLAIR) images mildly correlated (r = 0.6) with disease duration. No correlation could be observed between eVRS volume and number and the EDSS score. Male patients had a significant higher volume, but not number, of eVRS compare to female patients (p = 0.04). In the entire MS population, eVRS number mildly and inversely correlated with SPART*d* and DKEFS*fs* scores (r = -0.47 and r = -0.46, respectively). When RRMS and sPMS were merged and considered as a unique group, the volume of eVRS observed on 2DPSIR and 3DT1, but not on 3DFLAIR, significantly correlated with D-KEFS (r = -0.89, p = 0.002 and r = -0.83, p = 0.009, respectively). Moreover, a better correlation with eVRS number and D-KEFS was also obtained (2DPSIR: r = - 0.80, p = 0.016; 3DT1: r = -0-69, p = 0.05). Finally, no correlation was found between eVRS number and volume and BPF (r = 0.3), gadolinium-enhanced lesions (r = 0.2) and WM T2 lesion volume (r = 0.2).

## Discussion

Conflicting results have been published on frequency, volume and significance of eVRS in MS. [22] Achiron and Faibel (2002) detected a significantly higher number of eVRS in the convexity of the brains of patients with early MS (55%) compared to HC (7%). Wuerfel et al. [6], who analysed eVRS number and volume in 45 MS patients and 30 HC with a 1.5T scanner, reported no difference in VRS number between the two groups, both in the basal ganglia and in the cerebral high convexity bilaterally. However, these Authors observed a significantly association of eVRS volume with active WM lesions but not with whole brain atrophy, thus they suggested a possible role of eVRS as an inflammatory, rather then neurodegenerative, marker in MS. Furthermore, Conforti et al. [7] found an increase in dilated VRS in non active MS phases and a lack of correlation with the degree of global cerebral atrophy, as evaluated by means of FSL SIENA-X, suggesting a role for VRS as markers of inflammation-demyelination.

More recently, Etemadifar et al. [23] observed that the number of eVRS was significantly higher in patients with very early MS (i.e., <three months from clinical onset) compared to age- and sex-matched HC (p<0.001). eVRS were significantly more located in the high convexity areas in the MS group (p<0.001), while there was no significant difference in basal ganglia and midbrain VRS numbers. However, the number of eVRS with the size >2mm were more frequently observed in MS than in HC. With regard to the shape, round-shaped eVRS were significantly more frequent in MS, curvilinear shapes were significantly more frequent in HC, while no significant differences for oval shaped VRSs was observed between the two groups. By means of a high field (7T) scanner, Kilsdonk et al. [16] observed that he number of eVRS in MS patients was related to supratentorial brain volume fraction (BVF), age and disease duration, but not to WM lesion load. In HC, no association of eVRS count with age or supratentorial BVF was observed. On the base of these observations, eVRS were suggested to be more related to neurodegeneration than to inflammation. Certainly, literature data are quite conflicting and not comparable.

By means of a 3T MRI scanner we found that 2DPSIR was superior to both 3DT1 and 3DFLAIR in evaluating both number and volume of eVRS. Based on 2DPSIR images, we observed that male patients had higher eVRS volume, a finding in line with previous literature not only in MS, but also in HC and in people with dementia. Indeed, Ramirez et al. [24] found that demented and non-demented men had greater VRS in the white matter compared to women (p<0.001). In the study recently published by The Uniform Neuro-Imaging of Virchow-Robin Spaces Enlargement (UNIVRSE) consortium [25], aimed at harmonizing rating and analysis of VRS, male gender was found to be one of the risk factors for eVRS. Thus, compared to women, men seem to be more susceptible to greater volumes of VRS, particularly in the WM. In the study of Chen et al., [12], eVRS number increased with leukoaraiosis, atrophy, and advanced age (p<0.001). Moreover, individuals with Alzheimer’s disease or mild cognitive impairment had more eVRS than HC, and eVRS discriminated individuals with AD from those cognitively normal with an accuracy of 0.79 (95% CI, 0.69–0.89). In our study, we did not find an association with age, but it has to be stressed that the mean age of our cohort of patients was significantly lower compare to those of the cohorts of patients and HC analysed in the above mentioned studies.

The most important finding of our study was the significant inverse correlation observed between eVRS volume and cognitive performance in MS patients. This correlation was mild when the entire group of patients was considered, but significantly increased when only RRMS and SPMS were merged and considered as a unique group in the analysis. This result is particularly worth of interest since it suggests that the loss of brain tissue associates to the enlargement of the VRS may have pathological implication. The possibility that PSIR could amplify (due to its superior GM/WM contrast) the signal coming from the perivascular vessels, thus making over-estimated both the number and the volume of eVRS, was carefully evaluated by an accurate analysis of the HC brain, where eVRS were extremely rare also on 2DPSIR images.

It’s has to be stressed that the great majority of eVRS are visible only on 2DPSIR images but not on 3DT1 and, especially, on 3DFLAIR images. On 2DFLAIR images eVRS do not emerge from the apparently normal white matter. This suggests that a subtle, likely progressive, diffuse alteration in tissue integrity around vessels, beyond the resolution of conventional (T1 and FLAIR) MRI, occurs in MS. Thus, it might be possible that eVRS contribute to the changes observed in the so-called ‘normal appearing white matter’ (NAWM) with other unconventional MRI sequences. Indeed, the hypointensity of eVRS clearly reflect the high content in extracellular fluid that may account for the increase in diffusivity and the decrease in anisotropy observed in apparently non-lesional NAWM [26, 27].

The increased number and, especially, volume of eVRS in MS could be a form of local tissue loss and therefore contribute to brain atrophy, as observed in the study of Kilsdonk et al. (2015) with high magnetic field MRI, where eVRS count was found to correlate with brain atrophy, one of the best predictors for clinical deterioration in MS [28, 29]. Our study confirms the lack of association of eVRS with MRI parameters of WM damage. The lack of association of eVRS with brain atrophy, is probably due to the high percentage of patients (about 50%) at clinical onset enrolled in the study.

From a methodological point of view, on the base of our findings and experience with PSIR, we warmly recommend the use of PSIR sequence to detect and estimate the number and volume of VRS. The recent availability of 3D PSIR (not available when this study was designed and initiated) will probably improve the quality in measuring the volume of eVRS. When evaluating eVRS in MS, the comparison of PSIR sequence with T1 and, especially, FLAIR sequences is mandatory. Moreover, all scan evaluation should be performed by at least two independent observers.

In summary, 2DPSIR allowed the identification of a greater number of eVRS in MS compared to 3DT1 and 3DFLAIR. eVRS were found to correlate with the impairment in two cognitive tests, namely SPART*d* and DKEFS*fs*. Further studies in larger number of patients will confirm whether eVRS have a clinical significance and can be interpreted, in a broader sense, as markers of neurodegeneration in MS.
